# Patterns of Brain Activation and Meal Reduction Induced by Abdominal Surgery in Mice and Modulation by Rikkunshito

**DOI:** 10.1371/journal.pone.0139325

**Published:** 2015-09-30

**Authors:** Lixin Wang, Sachiko Mogami, Seiichi Yakabi, Hiroshi Karasawa, Chihiro Yamada, Koji Yakabi, Tomohisa Hattori, Yvette Taché

**Affiliations:** 1 CURE/Digestive Diseases Center and Center for Neurobiology of Stress, Department of Medicine, Digestive Diseases Division, University of California at Los Angeles, and VA Greater Los Angeles Health Care System, Los Angeles, California, United States of America; 2 Tsumura Research Laboratories, Kampo Scientific Strategies Division, Tsumura & Co., Ibaraki, Japan; 3 Department of Gastroenterology and Hepatology, Saitama Medical Center, Saitama Medical University, Saitama, Japan; Charité-Universitätsmedizin Berlin, Campus Benjamin Franklin, GERMANY

## Abstract

Abdominal surgery inhibits food intake and induces c-Fos expression in the hypothalamic and medullary nuclei in rats. Rikkunshito (RKT), a Kampo medicine improves anorexia. We assessed the alterations in meal microstructure and c-Fos expression in brain nuclei induced by abdominal surgery and the modulation by RKT in mice. RKT or vehicle was gavaged daily for 1 week. On day 8 mice had no access to food for 6–7 h and were treated twice with RKT or vehicle. Abdominal surgery (laparotomy-cecum palpation) was performed 1–2 h before the dark phase. The food intake and meal structures were monitored using an automated monitoring system for mice. Brain sections were processed for c-Fos immunoreactivity (ir) 2-h after abdominal surgery. Abdominal surgery significantly reduced bouts, meal frequency, size and duration, and time spent on meals, and increased inter-meal interval and satiety ratio resulting in 92–86% suppression of food intake at 2–24 h post-surgery compared with control group (no surgery). RKT significantly increased bouts, meal duration and the cumulative 12-h food intake by 11%. Abdominal surgery increased c-Fos in the prelimbic, cingulate and insular cortexes, and autonomic nuclei, such as the bed nucleus of the stria terminalis, central amygdala, hypothalamic supraoptic (SON), paraventricular and arcuate nuclei, Edinger-Westphal nucleus (E-W), lateral periaqueduct gray (PAG), lateral parabrachial nucleus, locus coeruleus, ventrolateral medulla and nucleus tractus solitarius (NTS). RKT induced a small increase in c-Fos-ir neurons in the SON and E-W of control mice, and in mice with surgery there was an increase in the lateral PAG and a decrease in the NTS. These findings indicate that abdominal surgery inhibits food intake by increasing both satiation (meal duration) and satiety (meal interval) and activates brain circuits involved in pain, feeding behavior and stress that may underlie the alterations of meal pattern and food intake inhibition. RKT improves food consumption post-surgically that may involve modulation of pain pathway.

## Introduction

Reduced appetite is one of the symptoms occurring after surgery, which can involve altered gut and brain signals induced by postoperative ileus and pain [1;2]. Following abdominal surgery, peripheral signals including those relayed by capsaicin sensitive afferent fibers send information to the brain thereby initiating changes in brain neuronal activity and release of neuropeptides [3–5], which may inhibit appetite and feeding behavior [6]. Ghrelin, insulin and leptin are implicated in the control of food intake and associated with eating disorders [7]. We showed in rats that abdominal surgery induces a rapid decline in plasma levels of acyl-ghrelin and food intake [6;8]. The hypothalamus and brainstem contain responsive nuclei to abdominal surgery known to regulate food intake as shown by the induction of c-Fos immunoreactivity, a marker of neuronal activity in rats [2;4;5;7–9].

Prolonged hospitalization due to postoperative ileus is a substantial burden to healthcare system [1;10;11]. Few of the existing strategies are sufficient to improve postoperative symptoms [1;11]. Clinical evidence indicates that early diet can reduce the risk of developing complications, and help the recovery [10;12]. Recently, agonists of ghrelin, a gut hormone that stimulates appetite and gastrointestinal motility, showed potential to treat postoperative ileus [13;14]. Unexpectedly, the clinical trials with TZP-101 was discontinued due to the failure to meet the endpoints, which may be related to the pharmacokinetic properties of this agonist [15]. Rikkunshito (RKT), is an herb extract of a Japanese Kampo medicine based on Chinese empirical recipe [16] reported to have beneficial effects on gastrointestinal dysfunction and anorexia [16;17]. RKT acts as a ghrelin enhancer [17;18] to alleviate gastroparesis [19;20], functional dyspepsia [16;19–21], and post-operative gastric ileus [22–24] in experimental and clinical studies. In our previous study, we demonstrated that RKT blocked L-dopa-inhibited gastric emptying in rats [25]. RKT can also restore novelty stress-induced reduction of food intake in mice [26–28].

To gain insight to mechanisms of food intake reduction induced by abdominal surgery, we assessed the alterations of meal pattern using automated feeding monitoring system, and mapped brain neuronal activation from the prefrontal cortex to medulla using immunohistochemistry for the early gene product, c-Fos in mice. We also investigated whether RKT administered orogastrically (og) would influence food intake, meal pattern, gastric emptying, plasma metabolic hormone levels and brain c-Fos expression altered by abdominal surgery. Changes in catecholaminergic neurons in the ventrolateral medulla (VLM) and nucleus tractus solitarius (NTS) known to be activated by abdominal surgery in rats [3;5;29;30] were also examined by double-labeling with tyrosine hydroxylase (TH) in mice.

## Material and Methods

### Animals

Adult male mice C57BL/6 (9–10 weeks-old, body weight 23–28 g, Harlan, San Diego, CA) quarantined for one week after arrival to facilities were maintained group-housed (4/cage) with an enriched environment under conditions of controlled temperature (21–23°C) and light cycle (6:00 AM– 6:00 PM). Mice were fed *ad libitum* with standard rodent chow (Prolab RMH 2500; PMI Nutrition International, Inc., Brentwood, MO, USA) and water. Experimental procedures followed NIH guidelines and were undertaken under the auspices of an OLAW Assurance of Compliance (A3002-01) and approved Animal Components of Research Protocols (IACUC Committee of the VA Greater Los Angeles Healthcare System, #01002–12).

### Compounds

Rikkunshito in powder form contained Atractylodis lanceae rhizoma (4 g, 18.6%), Ginseng radix (4 g, 18.6%), Pinelliae tuber (4 g, 18.6%), Poria (4 g, 18.6%), Zizyphi fructus (2 g, 9.3%), Citri Unshiu Pericarpium (2 g, 9.3%), Glycyrrhizae radix (1 g, 4.7%), and Zingiberis rhizoma (0.5 g, 2.3%) (Tsumura & Co., Tokyo, Japan). The compound was kept at room temperature and suspended in water before each experiment.

### Abdominal surgery

Mice which had no access to food for 6–7 h between 10 AM and 5 PM were anesthetized with isoflurane in O_2_ (3.5%). After a median laparotomy (about 1.5 cm), the cecum was exteriorized, placed in saline-soaked gauze and manipulated between two fingers for 1 min. Thereafter, the cecum was placed back into the abdominal cavity and the peritoneum, muscle layers and skin were sutured. Anesthesia and surgery lasted for approximately 10 min and mice regained the righting reflex within 2–3 min after removal of the vapor anesthesia. Controls were remained in their home cages without access to food or water. Stress other than caused by surgery was minimized by acclimating mice to the experimental conditions and avoiding transport. We did not include mice exposed to isoflurane alone as controls because anesthesia is also an intrinsic component of abdominal surgery.

### Automated monitoring of meal microstructures

Feeding behavior was monitored using the BioDAQ episodic feeding monitoring system for mice (BioDAQ, Research Diets, Inc., New Brunswick, NJ) as detailed in our previous studies [31;32]. This allows us to monitor continuously meal patterns in undisturbed mice with minimal human interference. Mice were habituated for one week to single housing and fed on AIN-93M rodent diet (Research Diets, Inc.) through a feeding hopper inserted onto regular housing cages containing environmental enrichment and bedding material. The AIN-93M is a balanced rodent diet less prone to spillage during eating and therefore insured more accurate measurements of food intake. Water was provided *ad libitum* from regular water bottles. Data were collected over a 24 h period.

The microstructure analysis of food intake was based on the manufacturer’s recommendations as validated in our previous studies [31]. The BioDAQ system weighs the hopper with food at the ±0.01 g level of accuracy second by second and algorithmically detects 'not eating' as weight stable and 'eating' as weight unstable. Feeding bouts (changes in stable weight) are recorded as feeding bout vectors with a start time, duration and amount consumed. Bouts are separated by an inter-bout interval, and meals consist of one or more bouts separated by an inter-meal interval. The inter-bout interval, inter-meal interval and minimum meal amount are user definable and were set at 5 sec, 5 min and 0.02 g respectively. Thus, food intake was considered as one meal when the feeding bouts occurred within 5 min of the previous bout and their sum was equal to or greater than 0.02 g. When feeding bouts were longer than 5 min apart, they were considered as a new meal. Meal structures per time periods included the number of meals (meal frequency), meal size, meal duration, inter-meal interval (time difference between the end of one meal and the initiation of the next one) and total time spent eating (time in min or % spent in feeding bouts or meals). These parameters were calculated by the software provided by the manufacturer (BioDAQ Monitoring Software 2.2.02). The satiety ratio was calculated as the average inter-meal interval divided by the average meal size, and the rate of ingestion was expressed as meal eating rate in mg/min.

### Gastric emptying of semi-liquid non-nutrient meal in mice

Gastric emptying was determined as in our previous study [33] in mice deprived of food but not water for 6–7 h during light phase. Mice had no access to food and water after the surgery and were gavaged with 0.3 ml of 1.5% methylcellulose (Sigma-Aldrich, St Louis, MO) and 0.5% phenol red (Sigma-Aldrich) and 20 min later, euthanized by cervical dislocation. The abdominal cavity was opened, gastric pylorus and cardia were clamped, the stomach removed, rinsed, placed into 15 ml of 0.1 N NaOH, homogenized for 30 s (Polytron; Brinkman Instruments, Westbury, NY). The suspension was settled for 1 h at room temperature and then 5 ml of the supernatant added to 0.5 ml of 20% trichloroacetic acid (w/v; Sigma-Aldrich). The samples were centrifuged at 3000 rpm at 4°C for 20 min. Three ml of the supernatant were mixed with 4 ml of 0.5 N NaOH. The absorbency of the sample (1 ml) was read at 560 nm (Shimadzu 260 spectrophotometer). Phenol red recovered from animals euthanized immediately after the administration of the methylcellulose/phenol red solution served as a standard (0% emptying). Percentage of emptying in the 20-min period was calculated as % emptying = (1- absorbance of test sample/absorbance of standard) × 100.

### Plasma metabolic hormone assay

For plasma hormone assay, blood was withdrawn from the heart in mice under isoflurane anesthesia, and added with ethylenediaminetetraacetic acid (EDTA-2K), Pefabloc, protease inhibitor cocktail (all from Sigma-Aldrich,) and dipeptidyl peptidase IV inhibitor (EMD, Millipore Billerica, MA). Whole blood was centrifuged at 10000×g for 3 min and plasma samples were collected and stored at -80°C until use. Plasma ghrelin, leptin and insulin levels were measured using active-ghrelin ELISA kit (Code No. 97751, Mitsubishi Chemical Medience Corporation, Tokyo, Japan), mouse leptin Assay Kit (Code No. 27160, Immuno-Biological Laboratories Co., Ltd., Gunma, Japan), and Lbis® Insulin-Mouse-T (Code No. AKRIN-011T, SHIBAYAGI Co., Ltd., Gunma, Japan).

### Immunohistochemistry

Mice were deeply anaesthetized with isoflurane and perfused transcardially with saline followed by ice-cold fixative (4% paraformaldehyde and 14% saturated picric acid in 0.1 M phosphate buffer). Brains were removed, post-fixed overnight and cryoprotected in 10% sucrose for 1–2 days. Frozen coronal sections (30 μm) of the brain (from bregma: 1.98 mm to −8.00 mm), according to Franklin and Paxinos’s mouse brain atlas [34] were cut in a cryostat (Microm International GmbH, Walldorf, Germany). The immunoreactivity for c-Fos or tyrosine hydroxylase (TH) was detected by the avidin-biotin-peroxidase complex (ABC) immunohistochemical technique as previously described in mice [35].

For c-Fos-ir, free-floating sections were incubated for two days with rabbit polyclonal anti-c-Fos antibody (1:10,000, PC38, EMD Millipore). Then, sections were incubated for 1 h at room temperature with biotinylated secondary goat anti-rabbit IgG (1:1000, Cat No. 111-067-003; Jackson ImmunoResearch Laboratories Inc. West Grove, PA) followed by the ABC, (1:200; Vector, Burlingame, CA) for 1 h. The chromogen was diaminobenzidine tetrachloride (DAB, 0.025%, Sigma-Aldrich) with hydrogen peroxide (0.01%, Sigma-Aldrich).

For double labeling of c-Fos and TH in the medulla, sections were processed first for c-Fos using the same procedures as above except 2 modifications. First, the biotinylated secondary goat anti-rabbit IgG was a Fab fragment (1:2,000; Jackson ImmunoResearch Laboratories). Second, the DAB solution contained 2.5% nickel ammonium sulfate and 0.001% hydrogen peroxide in 0.1 M acetate buffer (pH 6.5). After thorough rinse, brain sections were incubated overnight with rabbit anti-TH antibody (1:2,000, MAB5280, EMD Millipore). The remaining processing was the same as for c-Fos.

Observation, cell counting and microphotography were performed using light microscopy (Axioscop II, Carl Zeiss, Germany). Immunoreactive cells were counted unilaterally except the area postrema. Quantified areas were chosen according to Franklin and Paxinos’ mouse brain atlas [34] in sections bearing the largest volume, therefore both ends with very small area in sections were not included. In the cortex, cells were counted inside a square (1 x 1 mm) of a grid in one of the ocular lenses with the 20x objective. In some nuclei with subdivisions, the portion or subnucleus with the most prominent increase in c-Fos expression was selected for counting, such as the ventrolateral bed nucleus of stria terminalis and external subnucleus of lateral parabrachial nucleus. Each area was counted in 4–5 sections. The average number of single or double labeled cells/section for each animal was calculated.

### Experimental protocols

RKT (0.5 g/kg) or vehicle (distilled water, 4 ml/kg) was given daily by orogastric gavage for 7 days, and on day 8, at 6 h and 1 h before the abdominal surgery that took place at 1–2 h before the dark phase. The last RKT treatment before surgery was to maintain the effect based on previous studies showing that RKT administered at 1 h before novelty stress in mice improves feeding [26–28]. Non-surgery controls remained in their home cages. Four sets of experiments were performed in different cohorts of mice treated with vehicle or RKT without or with abdominal surgery. (i) Food intake and meal pattern were monitored using BioDAQ system up to 24 h. (ii) Gastric emptying was measured at 2 h or 6 h post-surgery. (iii) Mice were anesthetized by isoflurane 2 h after the surgery and the blood was withdrawn from the heart for plasma hormones assay. (iv) Mice were perfused systemically by fixative 2 h after the surgery and brains were collected for immunohistochemistry.

### Statistical analysis

Data are shown as mean ± SEM. Comparison between multiple groups was performed by one-way ANOVA followed by Student-Newman-Keuls or Tukey post hoc multiple comparisons, and comparison between two groups by Student t-test. A P value < 0.05 was considered significant.

## Results

### Abdominal surgery inhibited food intake associated with increased satiation and satiety in mice

**Fig 1:**
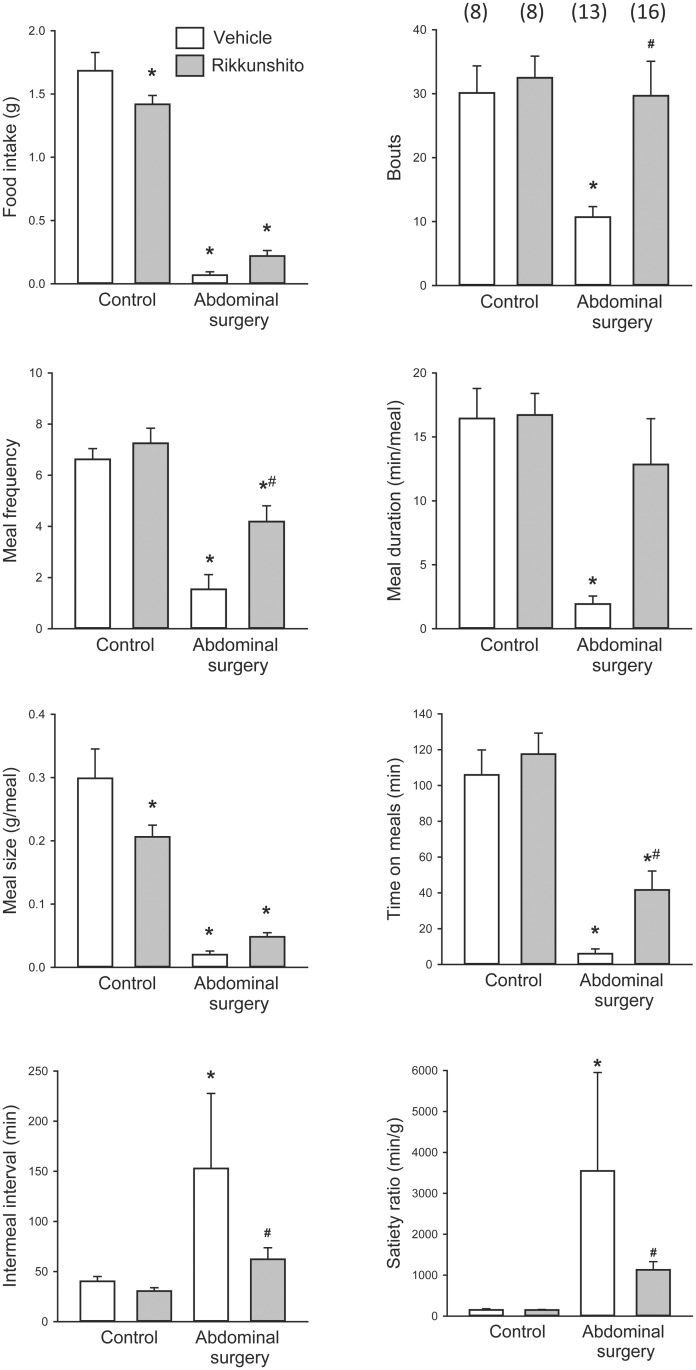
Alterations of meal patterns during the 6-h period post abdominal surgery in mice and influence of rikkunshito (RKT) pretreatment. Mice were gavaged with vehicle (distilled water) or RKT (0.5 g/kg) daily for 1 week, and on day 8 at 6 h and 1 h before surgery. Abdominal surgery (laparotomy and cecum palpation) was performed after 6–7 h food deprivation (from 10 AM to 4–5 PM). Nocturnal food intake was measured in an automated monitoring system (BioDAQ) during the first 6 h post-surgery. Data are mean ± SEM, the number of mice/group is indicated in parentheses; p < 0.05 *: vs. vehicle-control, #: vs. vehicle-abdominal surgery.

Daily pretreatment for one week with distilled water given by gavage followed by laparotomy and cecum palpation performed under short anesthesia in mice deprived of food during the light phase for 6–7 h, inhibited the cumulative food intake by 97%, 96%, 92% and 86% at 2, 6, 12 and 24 h post-surgery respectively compared with mice without surgery (Table 1, Fig 1). The meal pattern up to 12 h (mostly dark phase) after surgery still showed a significant reduction of feeding bouts, time spent on meals, and meal frequency, duration and size by 27%, 88%, 77%, 73% and 87%, respectively, and the intermeal interval (IMI) tend to be prolonged and the satiety ratio increased by 16.7 times (Table 1). Similar changes were present at 2 h (Table 1) and 6 h (Fig 1) and still maintained at 24 h post-surgery (Table 1).

**Table 1:** Abdominal surgery (AS) altered meal pattern in mice and partial prevention by oral rikkunshito (RKT) pretreatment.

		Food intake (g)	Bouts	Meal frequency	Total time on meals (min)	Meal duration (min)	Meal size (g)	IMI (min)	Satiety ratio (min/g)
2 h	V-C	0.71±0.13	13.6±1.9	3.4±0.4	50.2±4.3	15.4±1.4	0.21±0.03		
	RKT-C	0.60±0.05	15.3±1.6	3.5±0.5	60.0±8.9	19.7±4.6	0.18±0.02		
	V-AS	0.02±0.01*	6.5±1.3*	0.5±0.2*	2.2±1.0*	1.7±0.6*	0.02±0.01*		
	RKT-AS	0.12±0.03*	17.3±3.1^#^	1.9±0.3* ^#^	21.4±5.9* ^#^	13.7±5.0^#^	0.06±0.01*		
12 h	V-C	3.04±0.13	56.4±7.1	11.1±0.7	195.1±19.6	18.3±2.6	0.30±0.03	58.7±5.7	211.1±34.0
	RKT-C	2.61±0.07*	55.8±5.1	12.6±0.9	208.9±23.3	16.5±1.5	0.21±0.01	57.5±8.8	267.6±36.9
	V-AS	0.24±0.07*	15.0±1.8*	3.6±0.9*	22.5±5.9*	4.9±0.9*	0.04±0.01*	176.9±65.6	3745.1±1713.7*
	RKT-AS	0.49±0.08* ^#^	41.1±6.3^#^	7.6±0.9* ^#^	74.5±13.2* ^#^	9.9±2.0* ^#^	0.07±0.01*	104.7±20.2	1879.8±409.6
24 h	V-C	3.87±0.21	74.0±9.3	15.8±0.8	253.5±27.7	16.7±2.5	0.27±0.02	77.5±3.1	306.5±28.1
	RKT-C	3.13±0.13*	69.9±5.4	16.6±1.1	246.0±24.6	14.9±1.3	0.19±0.01*	68.9±8.2	356.6±35.8
	V-AS	0.56±0.16*	18.9±2.2*	5.8±1.2*	43.5±8.7*	7.7±0.8*	0.08±0.01*	190.7±62.7	2251.8±683.3*
	RKT-AS	0.90±0.11*	51.7±6.8^#^	11.6±1.3* ^#^	118.7±15.4* ^#^	10.2±1.3*	0.08±0.01*	111.2±14.3	1287.0±164.9* ^#^

V: vehicle, C: control (non-surgery). Data are mean ± SEM of n = 8/group in V-C and RKT-C, n = 13 in V-AS and n = 16 in RKT-AS; p < 0.05

*: vs. V-C,

^#^: vs. V-AS.

### RKT given orally as pretreatment improved food intake and meal pattern altered by abdominal surgery in mice

In RKT pretreated control (no surgery) mice without access to food during the light phase, the 12- and 24-h cumulative food intake was decreased by 14% and 19% respectively compared to vehicle pretreatment. This was associated with a reduced meal size, while no other parameters of meal structures were modified (Table 1, Fig 1). RKT treatment for one week before surgery partially, but significantly improved food intake at 12 h post-surgery, while at other time periods it did not reach significance by oOne-Way ANOVA (Table 1, Fig 1). Meal pattern analyses showed that in mice that underwent abdominal surgery, RKT improved meal structures leading to normalized feeding bouts at all-time points and meal duration up to 12 h (dark phase), and significantly increasing in meal frequency and size, and time spent on meals up to 24 h (Table 1 and Fig 1). RKT shortened IMI of the integrated 6 h meal pattern in mice with surgery (Fig 1), and lowered the satiety ratio at 6 as well as at 24 h post-surgery (Table 1).

### Abdominal surgery inhibited gastric emptying in vehicle and RKT treated mice

**Fig 2:**
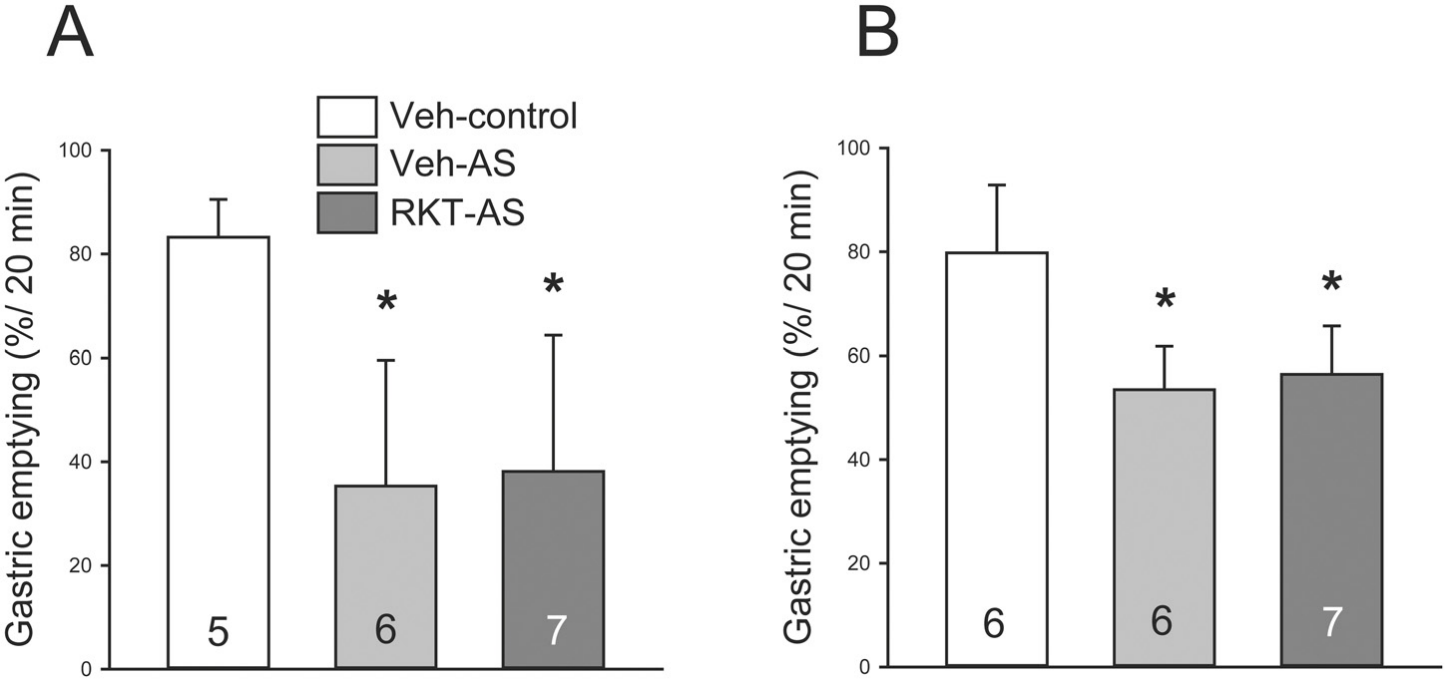
Abdominal surgery (AS) inhibited gastric emptying similarly in mice pretreated with vehicle or rikkunshito (RKT). Vehicle (distilled water) or RKT (0.5 g/kg) was administered by gavage daily for 1 week, and on day 8, before food deprivation and 1 h before surgery. Abdominal surgery (laparotomy and cecum palpation) was performed after 6–7 h food deprivation (from 10 AM to 4–5 PM). Gastric emptying of a non-nutrient viscous solution was determined during the 20 min period before the 2 h (A) or 6 h (B) post-surgery using phenol red-methylcellulose method. Data are mean ± SEM, and the number of mice/group is indicated in each bar; *: *p* < 0.05 vs. controls.

In mice treated with og water and deprived of food for 6–7 h during the light phase, abdominal surgery decreased gastric emptying compared to mice without surgery as assessed during the 20 min period before the 2 h (35.3 ± 9.9% vs. 83.3 ± 3.3%; *p* < 0.05; Fig 2A) and 6 h (53.5 ± 3.4% vs. 79.8 ± 5.3%, *p* < 0.05: Fig 2B) post-surgery. RKT (0.5 g/kg, og) daily for 1 week did not alter the delayed gastric emptying measured at either 2 (Fig 2A) or 6 h (Fig 2B) post-surgery (34.5 ± 11.0% or 56.5 ± 3.5% respectively).

### Abdominal surgery reduced plasma levels of acyl-ghrelin in vehicle and RKT pretreated mice

**Fig 3:**
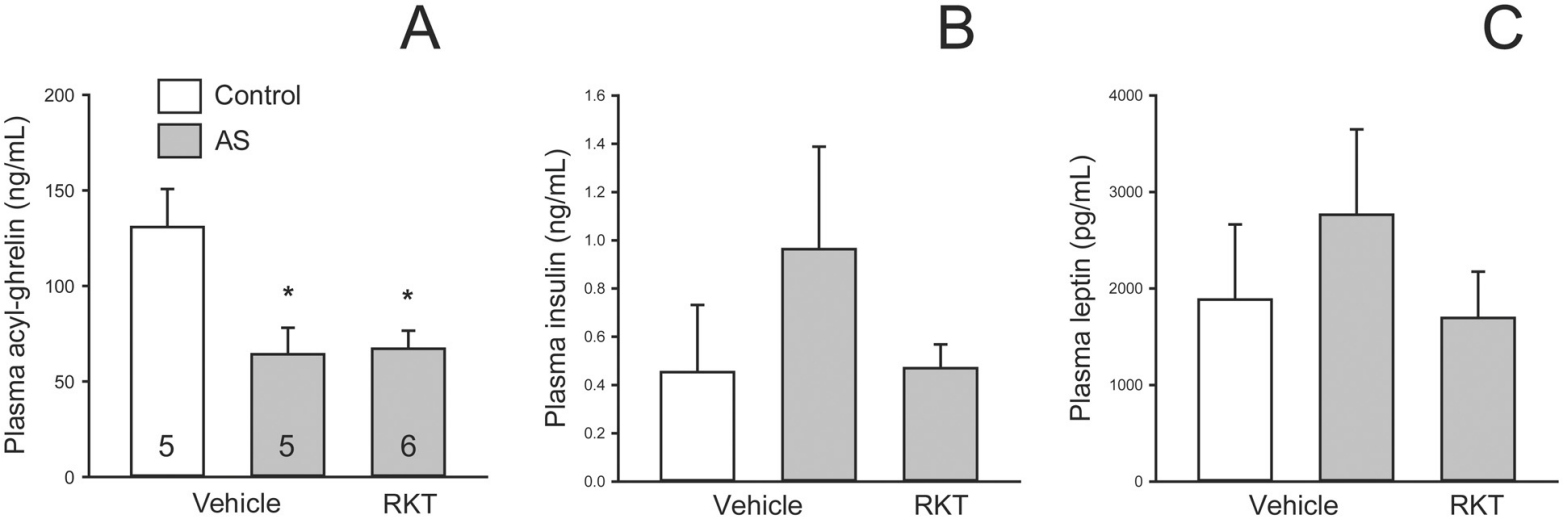
Abdominal surgery (AS) decreased plasma levels of acyl-ghrelin (A) in vehicle or RKT pretreated mice, while there was no significant difference in insulin (B) and leptin (C) levels among the treatments. Experimental conditions were similar as in Fig 2 legends, except mice were euthanized 2 h after the AS and blood was collected from the heart. Data are mean ± SEM; the number of mice/group is indicated in the graph A; *: *p* < 0.05 vs. controls.

In mice fasted for 6–7 h in the light phase, abdominal surgery significantly reduced plasma acyl-ghrelin levels compared to controls at 2 h post-surgery, and the response was not altered by RKT pretreatment (Fig 3A). There were no changes in plasma levels of insulin and leptin between the different treatment groups (Fig 3B and 3C).

### c-Fos immunoreactivity in the mouse brain induced by abdominal surgery and selective effect of RKT pretreatment

**Fig 4:**
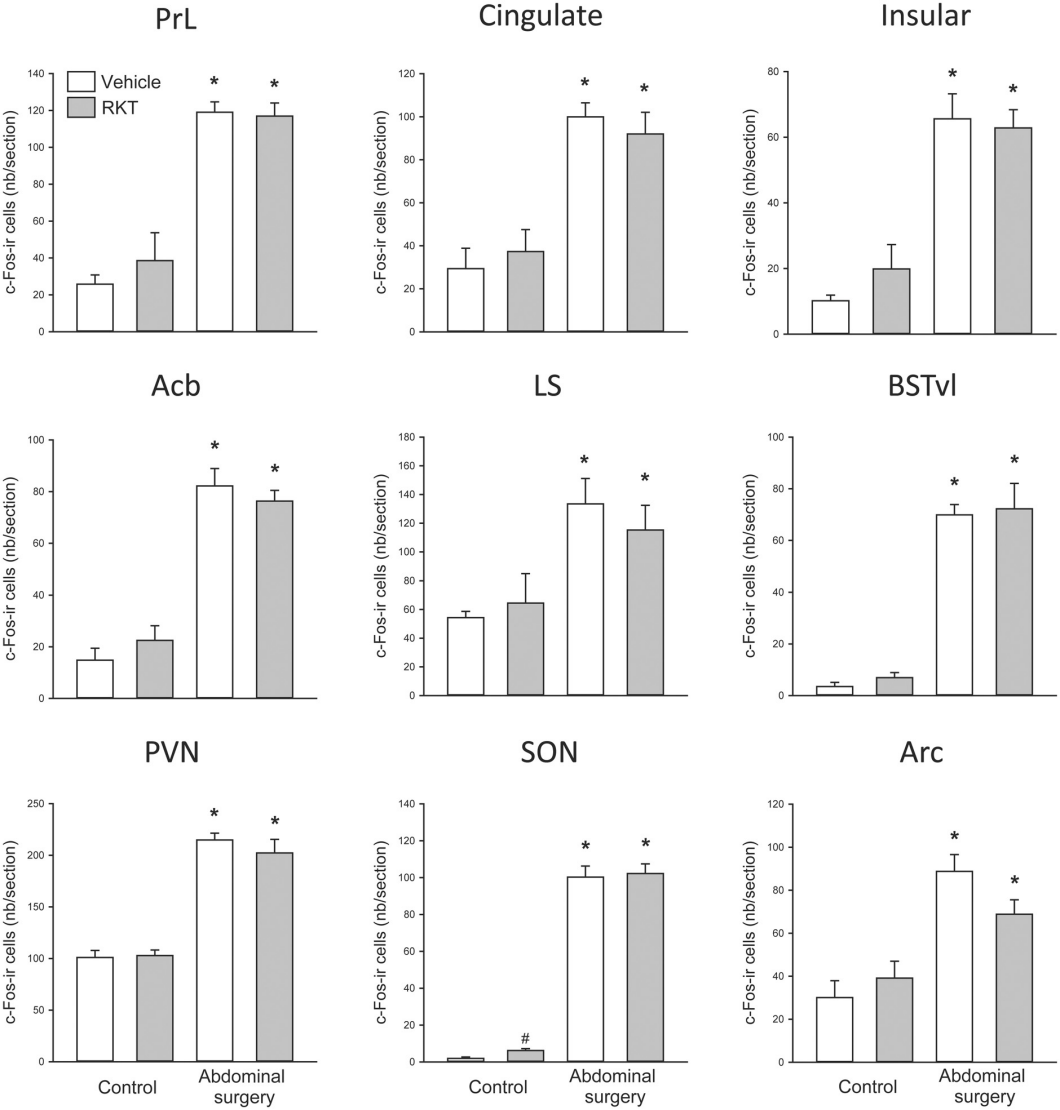
Effects of abdominal surgery and RKT pretreatment on the pattern of c-Fos immunoreactive (ir) cells in mice forebrain structures. Vehicle (distilled water) or RKT (0.5 g/kg) was administered by gavage daily for 1 week and on day 8, before the 6-7-h food deprivation during the light phase and 1 h before surgery. Abdominal surgery was performed 1–2 h before the onset of the dark phase. Mice were euthanized 2 h after the surgery. Data are mean ± SEM, n = 4-5/group; *: *p* < 0.05 vs. vehicle-control and #: *p* < 0.05 vs. vehicle-abdominal surgery. Acb: nucleus of accumbens; Arc: arcuate nucleus; BSTvl: ventrolateral subnucleus of bed nucleus of stria terminalis; LS: lateral septum; PrL: prelimbic cortex; PVN: paraventricular nucleus of the hypothalamus; SON: supraoptic nucleus.

**Fig 5:**
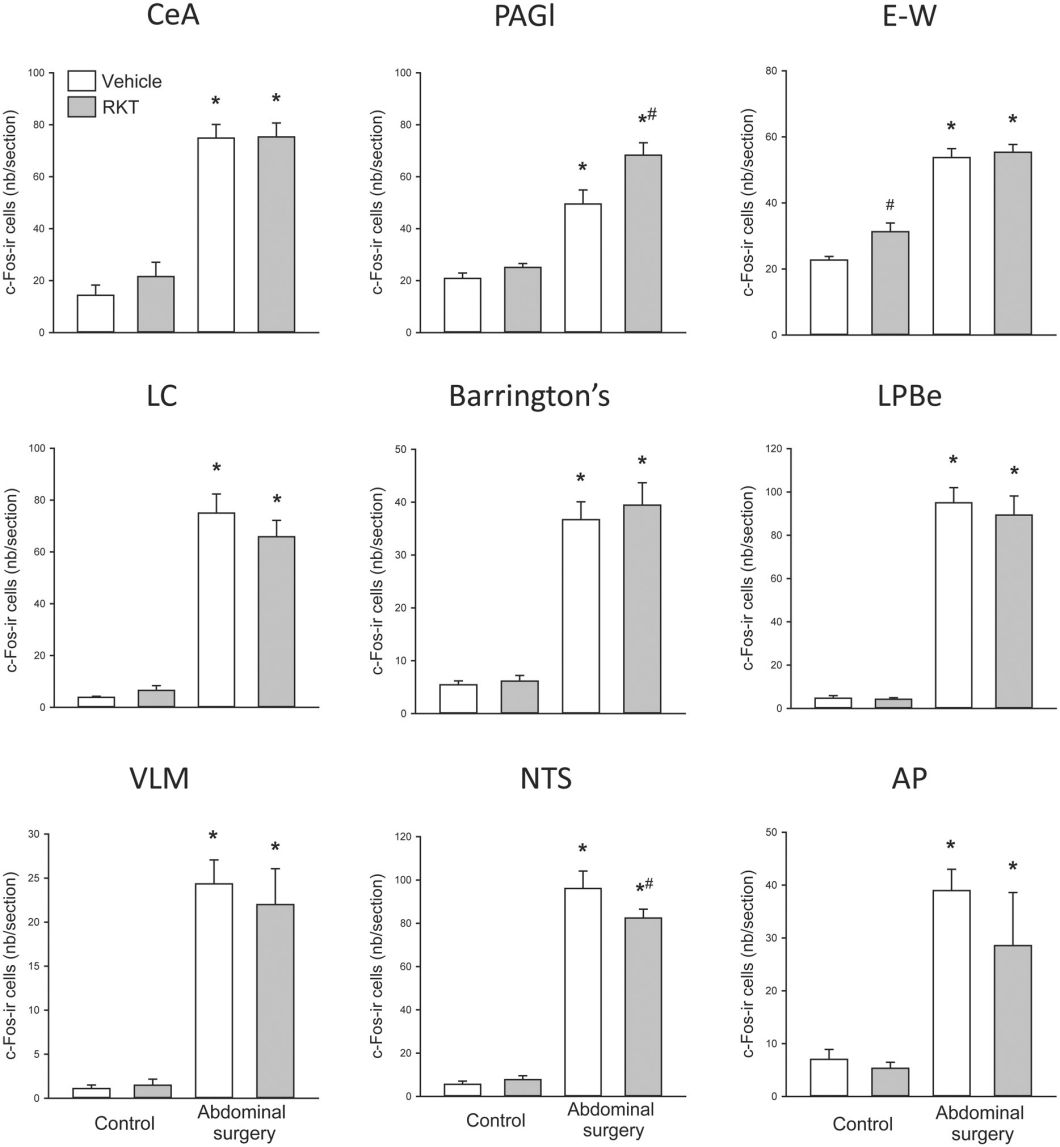
Effects of abdominal surgery and RKT pretreatment on the pattern of c-Fos immunoreactive (ir) cells in the central amygdala and brainstem structures in mice. Experimental conditions are as detailed in Fig 4 legends. Data are mean ± SEM, n = 4-5/group; *: *p* < 0.05 vs. vehicle-control and #: *p* < 0.05 vs. vehicle-abdominal surgery. AP: area postrema; CeA: central nucleus of the amygdala; E-W: Edinger-Westphal nucleus; LC: locus coeruleus; LPBe: external subnucleus of lateral parabrachial nucleus; NTS: nucleus tractus solitaries; PAGl: periaqueductal area, lateral area; VLM: ventrolateral medulla.

**Fig 6:**
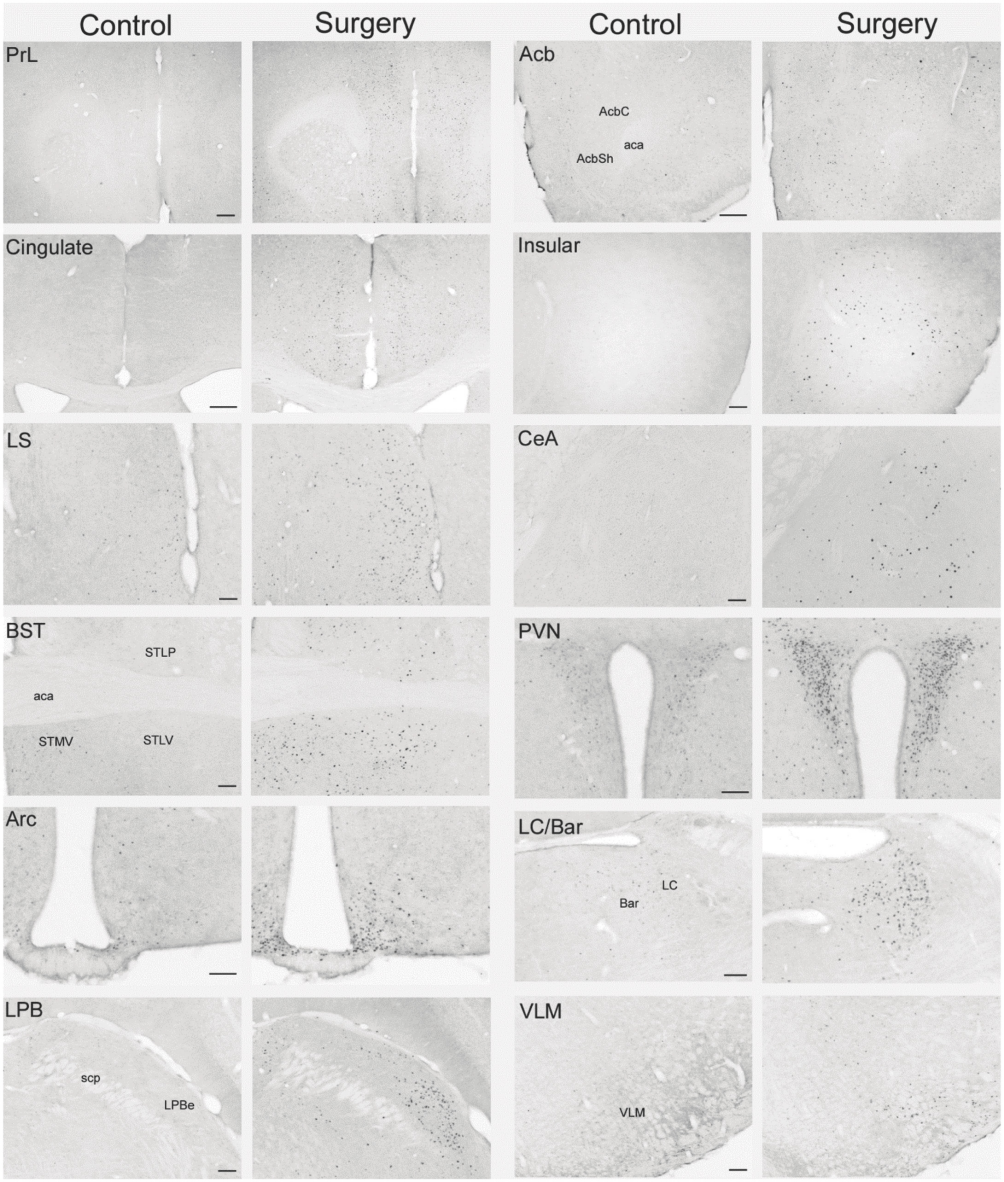
Representative photomicrographs of brain areas with increased c-Fos immunoreactivity induced by abdominal surgery in mice compared to controls (non-surgery). Experimental conditions are as detailed in Fig 4 except only vehicle pretreatment is shown. aca: anterior commissure, anterior part; Acb: nucleus accumbens; AcbC: accumbens nucleus, core; AcbSh accumbens nucleus, shell; Arc: arcuate nucleus; BST: bed nucleus of stria terminalis; CeA: central nucleus of the amygdala; Cg: cingulate cortex; LC/Bar: locus coeruleus/Barrington’s nucleus; LPBe: external subnucleus of lateral parabrachial nucleus; LS: lateral septum; PVN: paraventricular nucleus of the hypothalamus; PrL: prelimbic cortex; scp: superior cerebellar peduncle; STMV bed nucleus of the stria terminalis, medial division, ventral part; STLP: bed nucleus of the stria terminalis, lateral division, posterior part; STLV: bed nucleus of the stria terminalis, lateral division, ventral part; VLM: ventrolateral medulla. Scales = 100 μm.

**Fig 7:**
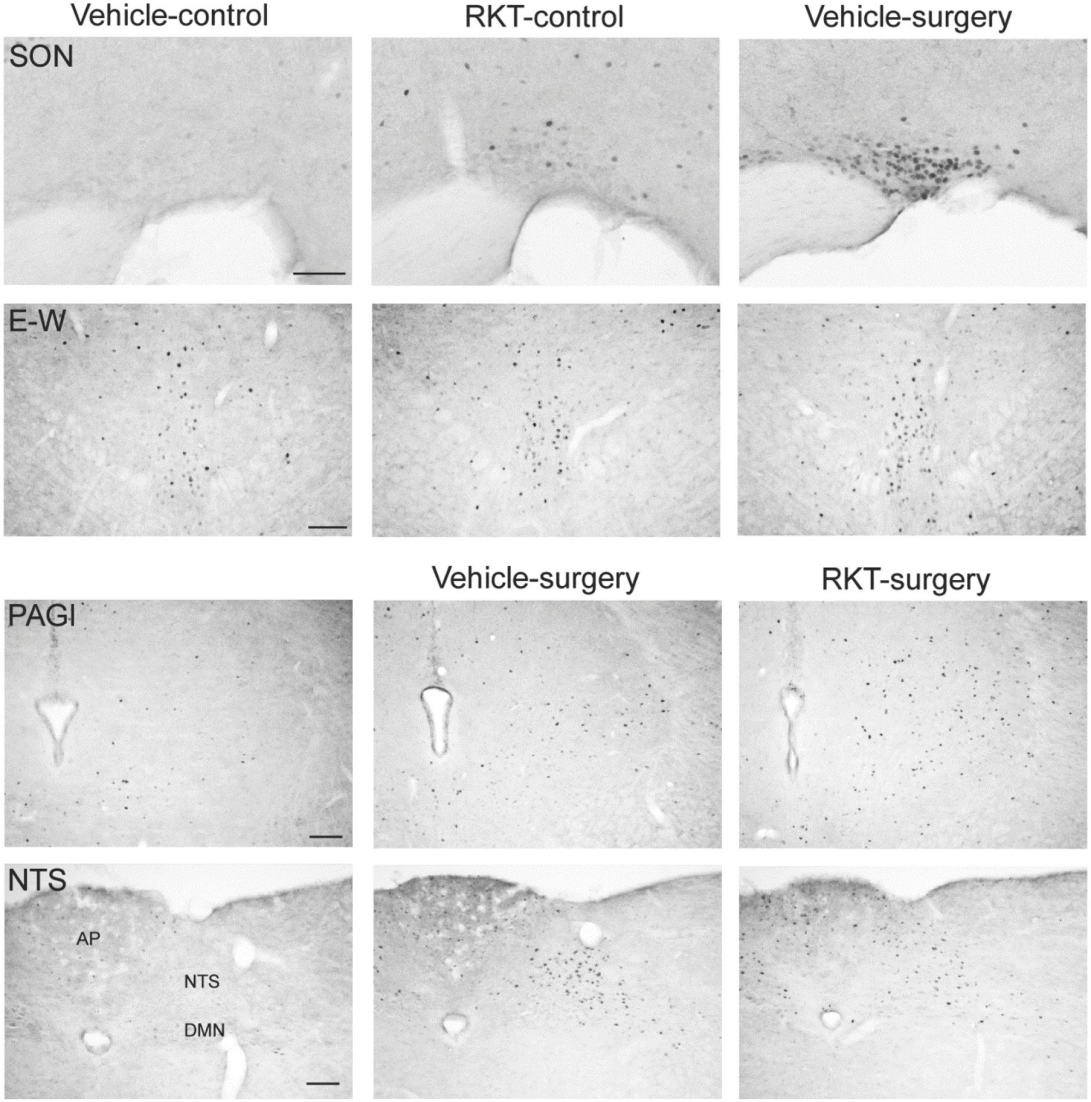
Photomicrographs of brain areas with c-Fos immunoreactivity significantly influenced by RKT pretreatment in mice with or without abdominal surgery. Experimental conditions are as detailed in Fig 4. AP: area postrema; DMN: dorsal motor nucleus of the vagus; E-W: Edinger-Westphal nucleus; NTS: nucleus tractus solitarius; PAGl: periaqueductal area, lateral area; SON: supraoptic nucleus. Scales = 100 μm.

In mice without access to food for 6–7 h in the light phase and euthanized around the onset of dark phase, og vehicle pretreatment without surgery induced moderate c-Fos-ir in some areas of forebrain and midbrain investigated, such as the prelimbic, cingulate and insular cortexes, lateral septum, paraventricular nucleus (PVN), arcuate nucleus (Arc), bed nucleus of stria terminalis (BST), periaqueduct gray (PAG) and Edinger-Westphal nucleus (E-W) (Figs 4–7). In mice pretreated with og vehicle, abdominal surgery increased significantly the number of c-Fos-ir cells/section compared to og vehicle and no surgery in the prelimbic (119.1 ± 5.6 vs. 25.8 ± 5.0), insular (65.6 ± 7.6 vs. 10.2 ± 1.7) and cingulate (99.9 ± 6.5 vs. 29.4 ± 9.5) cortexes, nucleus of accumbens (82.2 ± 6.8 vs. 14.8 ± 4.7), lateral septum (133.5 ± 17.7 vs. 54.4 ± 4.4), BST (ventral lateral subnucleus 69.9 ± 4.0 vs. 3.9 ± 1.6), central amygdala (74.9 ± 5.2 vs. 14.4 ± 3.9), supraoptic nuclei (SON; 100.3 ± 6.0 vs. 1.9 ± 0.8), PVN (214.8 ± vs. 101.1 ± 6.9), Arc (88.8 ± 7.9 vs. 30.1 ± 7.8), E-W (53.7 ± 2.7 vs. 22.7 ± 1.1), PAG lateral area (49.5 ± 2.1 vs. 20.9 ± 2.1), external subnucleus of lateral parabrachial nucleus (95.0 ± 7.0 vs. 4.7 ± 1.2), locus coeruleus (75.0 ± 7.4 vs. 3.9 ± 0.4), Barrington’s nucleus (36.7 ± 3.4 vs. 5.4 ± 0.7), A5 (data not shown), ventrolateral medulla (VLM; 24.4 ± 2.7 vs. 1.1 ± 0.4), nucleus tractus solitarius (NTS; 96.1 ± 8.0 vs. 5.6 ± 1.5) and area postrema (39.0 ± 4.1 vs. 7.0 ± 1.9) (Figs 4–7).

In control mice, compared to vehicle, RKT treatment increased the numbers of c-Fos-ir neurons in the SON (6.2 ± 1.0 vs. 1.9 ± 0.8 cells/section) and E-W (31.3 ± 2.6 vs. 22.7 ± 1.1; Figs 4, 5 and 7). In mice with abdominal surgery, RKT induced a significant increase in c-Fos-ir in the lateral PAG (68.3 ± 4.8 vs. 49.5 ± 5.4) and decrease in the NTS (82.4 ± 4.1 vs. 96.1 ± 8.0; Figs 5 and 7).

### Increased c-Fos and TH double-labeled neurons in the NTS and VLM induced by abdominal surgery were not altered by RKT pretreatment in mice

**Fig 8:**
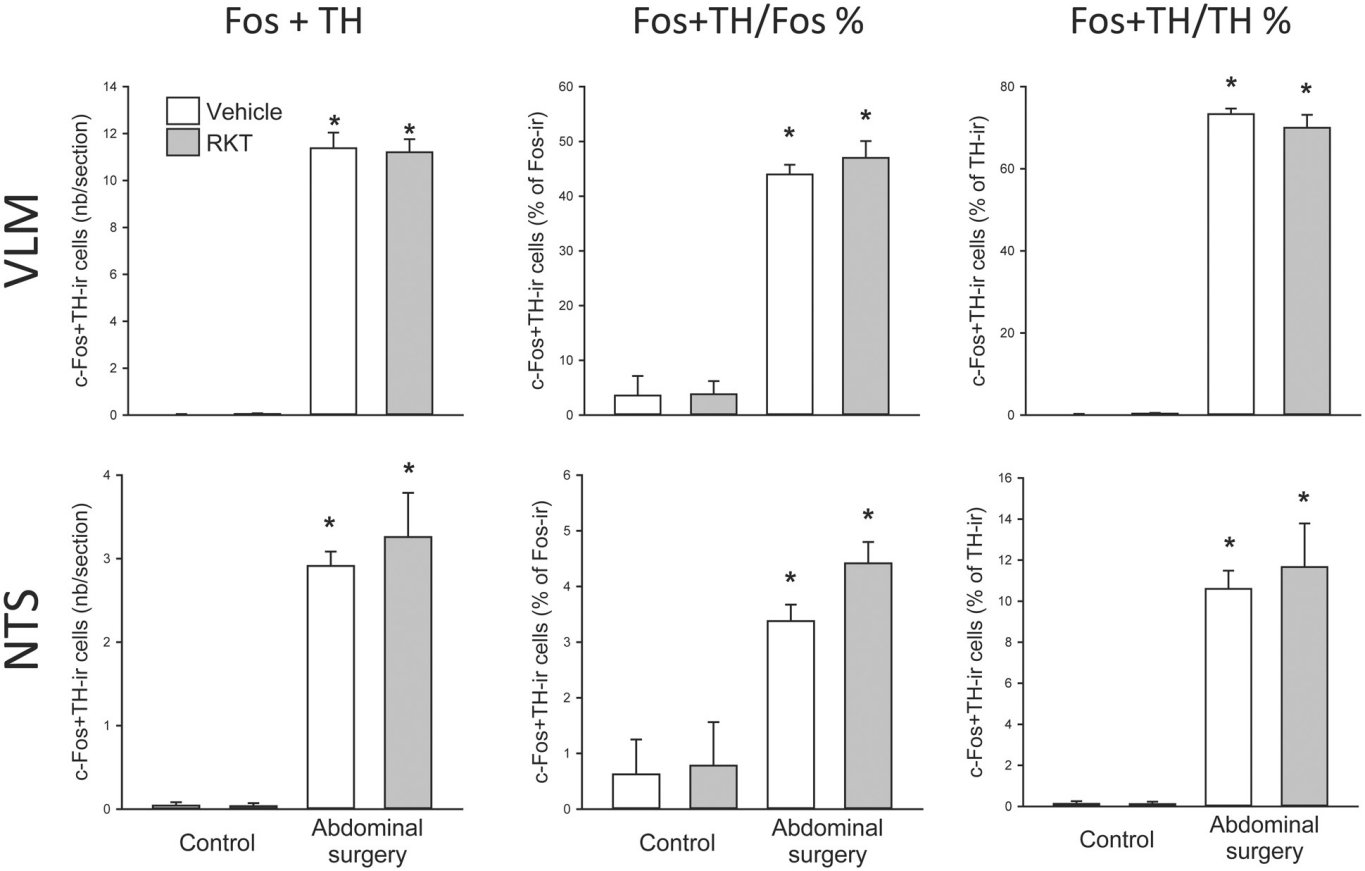
Cell counting of c-Fos and tyrosine hydroxylase (TH) double-immunoreactive (ir) neurons. in the nucleus tractus solitarius (NTS) and ventrolateral medulla (VLM) of mice pretreated with RKT. Experimental conditions are as detailed in Fig 4 legends. Data are mean ± SEM, n = 4-5/group; *: *p* < 0.05 vs. vehicle-controls.

Abdominal surgery induced significant higher numbered c-Fos-ir and c-Fos/TH-ir neurons compared to non-surgery in the NTS and VLM of mice pretreated with vehicle, without change in the number of TH-ir neurons (Table 2). However, the numbers of neurons with c-Fos-ir, TH-ir or c-Fos/TH-ir in the NTS and VLM were not significantly altered by RKT compared to vehicle pretreatment with or without surgery (Table 2). The percentage of TH/c-Fos double-labeled neurons induced by surgery compared to controls was high in the VLM (44.0 ± 1.8% vs. 3.6 ± 3.6% of c-Fos-ir and 73.3 ± 1.3% vs. 0.2 ± 0.2% of TH-ir neurons; Fig 8). In the NTS, the percentage of double-labeled neurons induced by surgery was significantly higher than controls (3.4 ± 0.3% vs. 0.6 ± 0.6% of c-Fos-ir and 10.6 ± 0.9% vs. 0.1 ± 0.1% of TH-ir neurons; Fig 8), although the percentage was low.

**Table 2:** c-Fos and TH double immunolabeling in the nucleus tractus solitarius (NTS) and ventromedial medulla (VLM) of mice at 2-h post abdominal surgery (AS) and pretreated with daily rikkunshito (RKT) for one week.

		Vehicle-C	RKT-C	Vehicle-AS	RKT-AS
NTS	c-Fos	3.6 ± 1.9	6.0 ± 0.6	88.2 ± 8.9*	73.6 ± 8.6*
	TH	27.7 ± 1.7	28.7 ± 1.8	27.7 ± 0.8	28.4 ± 0.8
	c-Fos+TH	0.0 ± 0.0	0.0 ± 0.0	2.9 ± 0.2*	3.3 ± 0.5*
VLM	c-Fos	0.8 ± 0.3	1.2 ± 0.3	25.9 ± 1.2*	24.0 ± 0.7*
	TH	13.7 ± 0.3	15.7 ± 1.0	15.6 ± 1.1	16.1 ± 1.1
	c-Fos+TH	0.0 ± 0.0	0.1 ± 0.0	11.4 ± 0.7*	11.2 ± 0.6*

Data are mean ± SEM of cells/section, n = 4-5/group;

*: *p* < 0.05 vs. Vehicle-C or RKT-C.

C: control (no surgery).

## Discussion

Abdominal surgery (laparotomy and cecum palpation under anesthesia) in mice without access to food for 6–7 h in the light phase induced a sustained 97–86% inhibition of cumulative food intake assessed from 2 to 24 h post-surgery periods compared to non-surgery controls. The meal pattern analyses revealed that the surgery induced both satiation (shorter meal duration and smaller meal size) and satiety (spent less time on meals, less bouts and meal frequencies, prolonged IMI and increased satiety ratio). This represents the first study that characterized alterations of meal pattern postoperatively in rodents. Most previous studies measured the gastric and/or intestinal motility and transit in rodent models of postoperative ileus [36–40], and only a few have assessed food intake mainly in rats [8;41;42]. The detailed information on alterations of meal structures in animal models is useful for testing new feeding interventions, particularly in the context that nutrient intake is beneficial for post-surgical recovery [10;12]. In a rat model, feeding increased gastric motility after abdominal surgery [41].

RKT daily pretreatment for one week in mice significantly improved the 12-h cumulative food intake compared to vehicle post-surgery, although the magnitude was small and the changes in food intake at other time points did not reach significance. Likewise, RKT induced only a modest amelioration of food intake in mice with chemical-induced acute lung injury [43]. However, meal structure analysis indicate that RKT increased appetite, as early as 2 h after the surgery and thereafter, with a significant increase in the numbers of bouts and meals, time spent on meals and meal duration, and shortened IMI and lowered satiety ratio compared to that in vehicle treated control mice. In particular, in mice with abdominal surgery, RKT normalized bouts and meal frequencies and duration to those in non-surgery group, indicative of increased drive to eat.

However, IMI and satiety ratio that are important indexes of suppressed appetite, were significantly shortened only in the 6 h meal pattern. This could result from (i) the weak effect of RKT on food intake and meal size; (ii) large individual differences in IMI in mice particularly those that underwent surgery in the vehicle group, and many mice with surgery did not have IMI in the early time points; and (iii) the cumulative 6 h monitored time encompassed the dark phase when mice eat the largest portion of their daily food [31]. Because of large individual differences in IMI, the satiety ratio calculated by dividing IMI by meal size also reflects the large variations inside treatment groups. This is a methodological issue that warrants further considerations taking into account that rodents do not ingest foods in regular meals while those are artificially defined for data analysis.

Loss of appetite, nausea and vomiting are manifestations of postoperative ileus [10;44;45]. Gastric emptying of a non-nutrient viscous solution was inhibited in mice by 58% and 45% from that of control as monitored during the 20 min period before the 2 and 6 h post-surgery, respectively. The basal 20 min gastric emptying value in the present experiment in mice was higher (80%) than in our previous studies (53%) [36;37]. This is likely related to the time of functional test performed in the early dark phase when mice start their nocturnal food intake and associated to the increased in cephalic vagal activity [46]. However, RKT pretreatment did not change the delayed gastric emptying of a viscous non-nutrient solution induced by surgery, which could be one of the factors for its limited effect on restoring the inhibited food intake. This suggests that RKT orexigenic effect is unrelated to changes in gastric emptying. In another mouse model of postoperative ileus, RKT recovered the delayed gastric emptying 24 h after the surgery [24]. The discrepancy between the present and previous studies could be related to differences in experimental conditions. Gastric emptying of solid meal was determined one day after a more invasive surgery (3–5 min compression of the terminal ileum), and one additional RKT treatment was performed 6 h after the surgery in the other study [24]. It is also possible that RKT effect is not potent enough to restore surgery-inhibited gastric emptying in early postoperative phase. In addition, its effect to improve the accommodation of the proximal stomach [21;47;48] may facilitate the retention of gastric content.

Along with delayed gastric emptying, abdominal surgery in mice decreased fasting plasma levels of acyl-ghrelin, as reported in our previous studies in rats [6] strengthening the inhibitory effect of abdominal surgery on circulating active form of ghrelin in rodents. By contrast, the plasma levels of insulin and leptin were not significantly modified by the surgery, which supports the selective alteration of plasma acyl-ghrelin post-surgery. We previously established in rats that this inhibition of ghrelin involved the activation of peripheral somatostatin 2 receptors [6]. Ghrelin is a potent gastrointestinal prokinetic and orexigenic peptide [13;49;50]. RKT is known as a ghrelin enhancer [18;51] that reverses decreased plasma levels of ghrelin in humans and animals treated with cancer chemotherapy drugs [52;53] or novelty stress [26;27]. In the present study, RKT did not change the decreased plasma levels of acyl-ghrelin determined at 2 h after the abdominal surgery in mice. This suggests that RKT effect to improve meal pattern in the mice is not related to the increase in plasma acyl-ghrelin levels at least in the early phase post-surgery although we cannot rule out that that RKT acts by enhancing ghrelin action at the receptor level [18;49].

Convergent studies in rats showed that abdominal surgery induces c-Fos expression in brain autonomic regulatory centers, such as the CeA, PVN, SON, E-W, locus coeruleus, NTS and VLM [4;5;29;30]. By contrast, in mice only two studies have assessed c-Fos induction post-surgery with a focus on the dorsal vagal complex [54;55]. Our mapping of c-Fos expression from the prefrontal cortex to the caudal medulla in mice at 2 h after the abdominal surgery indicates its occurrence in brain areas associated with stress, pain, feeding and regulation of visceral function. Namely, we found increases in Fos positive cells in the lateral septum, BST, CeA, PVN, locus coeruleus and VLM, which are the well-known brain centers responsive to visceral stress and being activated by corticotrophin-releasing factor (CRF) and norepinephrine (NE) signaling pathways [9;56–58]. The activation of the prelimbic, insular and cingulate cortexes, nucleus of accumbens and CeA could reflect the forebrain structures playing multiple roles in emotion control in response to pain and discomfort caused by the surgery [59]. Postoperative gastrointestinal dysfunction and suppressed appetite may also modulate neuronal activity in the NTS, LPB and Arc, which are involved in receiving visceral inputs and regulating gut motility and food intake [60–63]. Studies showed that c-Fos-ir occurred in the NTS neurons labeled by retrograde tracing from the small intestine in mice exposed to laparotomy and intestine palpation [54], indicative that NTS neurons are activated by intestine projecting neurons. The LPB known to receive taste and gastric inputs [64] is an important relay and integrative area between the NTS and rostral autonomic centers to influence ultimately feeding behavior [64–66]. The external subnucleus of LPB processes signals activated by various visceral stimuli [67–69]. The increased c-Fos-ir neurons induced by surgery in the Arc were mostly located in the dorsal lateral part that may be related to the activation of anorexigenic neurons containing pro-opiomelanocortin [70]. The E-W was also activated by abdominal surgery in rats [29] and likewise in mice (present study). This nucleus in the rodent brain contains the highest concentration of neurons expressing urocotin-1 [71], a member of CRF neuropeptide family that plays a role in stress-associated suppression of food intake [72;73].

The basal c-Fos expression is higher in some of the forebrain and midbrain structures, such as the lateral septum, PVN, Arc and E-W, compared to hindbrain nuclei. This may be induced by the 6–7 h food deprivation period before the dark phase. Indeed, in control mice, the c-Fos-ir was mostly located in the ventromedial area of the Arc, which is responsive to food deprivation [35;74–76] or appetite-stimulating peptides [50;75]. However, the magnitude of Fos expression at this site was low compared in overnight fasted mice [35]. In contrast, the hindbrain was very “quiet” under basal conditions of mice deprived of food during the light phase. This suggests that food deprivation during the light phase when rodents eat small portion of their daily food intake has less influence in the hindbrain than the forebrain.

Compared to vehicle, RKT treatment in control mice induced a small but significant increase in c-Fos-ir neurons in the SON and E-W (average 4 neurons per section in the SON and 9 in the E-W). Both nuclei bear urocortin-1-containg neurons [71], a peptide well established to play a role in stress-related inhibition of food intake [73] and stress adaptation [72]. RKT was shown to restore novelty stress-inhibited food intake in mice [26;28] and the anorexic effect of urocortin 1 injected intracerebroventricularly [77]. Whether urocortin-1 is modulated in the E-W and SON by RKT treatment needs to be further investigated.

In mice undergoing surgery, RKT induced a small but significant increase in the number of c-Fos-ir cells in the lateral PAG and decrease in the NTS. There is evidence showing that the PAG is part of the pain inhibitory descending pathways to the medulla and spinal cord [78;79]. The NTS, a well-known brain nucleus regulating food intake, is also situated in the brain circuitry of pain control [80], and has bi-directional connections with the PAG [81]. Therefore, the RKT-induced modulation of c-Fos expressing neurons in these brain nuclei are likely playing a role in feeding behavior in response to pain, stress and discomfort caused by the surgery and postoperative ileus.

The VLM (A1/C1 area) contains epinephrine and norepinephrine neurons expressing abundant TH, the step-limiting enzyme in the synthesis of catecholamine [82;83]. We found a high percentage of TH-ir neurons double labeled with c-Fos in the VLM (73%), while it was low in the NTS (11%) of mice that received surgery. The VLM is the relay center from the PAG to spinal cord and involved in descending analgesic signaling [78] and also regulates sympathetic activity [84;85]. The prominent activation of catecholaminergic neurons in the VML induced by surgery supports the VLM as an important area responding to visceral stress and pain, that may play a role in the sympathetic nervous system activation in postoperative ileus [85].

As abdominal surgery involves anesthesia, laparotomy and gut manipulation, the major determinants contributing to the observed functional changes and brain neuronal activation cannot be delineated. However, studies in rats showed that anesthesia had little effect on c-Fos expression while surgery was the major determinants [5].

In conclusion, mice that received laparotomy and cecum palpation are a useful animal model to test therapeutic approaches to improve functional alterations induced by postoperative ileus. Beside the well-known inhibited gastrointestinal transit and food intake, the monitoring of meal pattern and brain circuits provide parameters to delineate whether the treatment improve feeding behavior at target pathways modulating feeding, stress and pain. RKT pretreatment is able to stimulate feeding behavior independently of the inhibited gastric emptying or circulating acyl-ghrelin and to modify brain c-Fos in selective areas involved in pain and food intake. Since RKT orexigenic effect is mild, it can be combined with other strategies to treat gastrointestinal motor dysfunction and anorexia associated with surgery [17].
